# Efficient hydrogen isotopologues separation through a tunable potential barrier: The case of a C_2_N membrane

**DOI:** 10.1038/s41598-017-01488-8

**Published:** 2017-05-03

**Authors:** Yuanyuan Qu, Feng Li, Mingwen Zhao

**Affiliations:** 1 School of Physics, Shandong University, Jinan, 250100 Shandong China; 2 School of Physics and Technology, University of Jinan, Jinan, 250022 Shandong China

## Abstract

Isotopes separation through quantum sieving effect of membranes is quite promising for industrial applications. For the light hydrogen isotopologues (eg. H_2_, D_2_), the confinement of potential wells in porous membranes to isotopologues was commonly regarded to be crucial for highly efficient separation ability. Here, we demonstrate from first-principles that a potential barrier is also favorable for efficient hydrogen isotopologues separation. Taking an already-synthesized two-dimensional carbon nitride (C_2_N-*h*2D) as an example, we predict that the competition between quantum tunneling and zero-point-energy (ZPE) effects regulated by the tensile strain leads to high selectivity and permeance. Both kinetic quantum sieving and equilibrium quantum sieving effects are considered. The quantum effects revealed in this work offer a prospective strategy for highly efficient hydrogen isotopologues separation.

## Introduction

The separation of hydrogen isotopologues, such as H_2_, D_2_ and T_2_, is a crucial and inevitable stage for their applications in the nuclear industry. However, the low productivity and high energy cost of conventional hydrogen isotopologues separation methods hamper the relevant applications^1–5^. The emerging quantum sieving technology for isotopes separation has seized great attention due to its low energy consumption. Most of the prevalent works focus on the adsorptive capacities or diffusion properties of isotopologues in specific confined systems, such as carbon nanotubes, carbon nanohorns, metal-organic framework, as well as single-atomic membranes^6–11^. These porous structures impose nano-confinement to the guest molecules, invoking difference in the quantum levels associated with different masses, which can be magnified at low temperature for isotopes separation^12^. The separation efficiency of these systems significantly depends on the pore size and shape^10, 13–16^. However, the precise control of the porous structures remains a challenge in experiments.

Previous works using the equilibrium and kinetic quantum sieving approaches indicate that the confinement of potential wells to isotopologues is favorable for H_2_/D_2_ separation^6, 7, 15–17^, whereas potential barriers are regarded to be unsuitable for efficient isotopologues separation due to its low permeance^10^. However, when a barrier exists on the diffusion path, quantum tunneling effect begins to impact the diffusion behaviors at low temperatures, especially for such light molecule as hydrogen. This phenomenon has been demonstrated to effectively enable the helium isotopes separation by several two-dimensional (2D) membranes at low temperatures, where the lighter isotope (e.g. ^3^He) is more inclined to transmit through the membranes^18–20^. In these systems, the interaction between the membrane and the guest isotopes can be tuned by applying tensile strain which modifies the pore size of the membrane, and highly efficient isotopes separation with optimized selectivity and permeance can be obtained^21^. It is noteworthy that the zero-point-energy (ZPE) of hydrogen in confined systems would affect the energy barrier on the diffusion pathway^12^. Heavier isotopologues (e.g. D_2_) experience a lower energy barrier than the light ones (e.g. H_2_) according to the inversely scale law of the vibrational frequencies versus masses, which favors the transmission of the heavier ones, in contrary to the quantum tunneling effect^22^. The ZPE effect has been investigated in the cylindrical confinement systems such as carbon nanotubes by using equilibrium quantum sieving approaches^6, 15, 16^. Additionally, quantum tunneling of hydrogen ions through a graphene sheet or a hexagonal boron nitride membrane offered alternatives for isotopes separation^23–25^.

**Figure 1:**
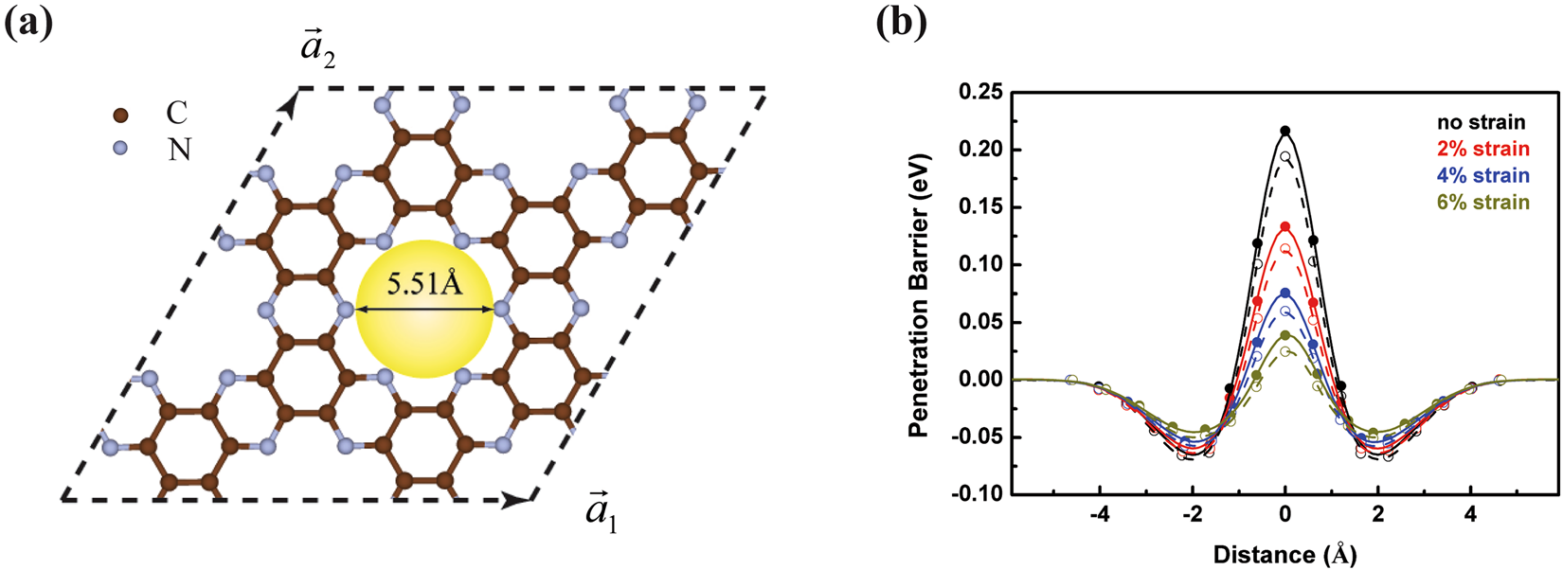
(**a**) The top view of the optimized (2 × 2) C_2_N-*h*2D supercell, where ${\overrightarrow{a}}_{1}$, ${\overrightarrow{a}}_{2}$ represent two primitive vectors. The brown and blue balls represent the C and N atoms respectively. The inscribed circle is indicated by the yellow circle. (**b**) The effective barriers of H_2_ (solid balls, solid lines), D_2_ (open balls, dashed lines) passing through C_2_N-*h*2D membrane along the perpendicular direction under different tensile strains. Colored points indicate the results obtained by first-principles calculations; while the curves show the numerically interpolated potentials.

In this work, we investigate the quantum tunneling of hydrogen isotopologues through an energy barrier by taking the ZPE correction into account. We employed kinetic quantum sieving and equilibrium quantum sieving approaches within a pseudo-one-dimensional approximation. Using an experimentally available membrane (C_2_N-*h*2D) as an example, we demonstrate that the competition between quantum tunneling effect and ZPE effect regulated by the tensile strain leads to high selectivity and permeance of hydrogen isotopologues separation. This provides a promising strategy for efficient D_2_ harvest for both scientific and industrial applications. In particular, we show that the unique porous structure of C_2_N-*h*2D with uniformly distributed pores (as shown in Fig. 1(a)), offers an ideal energy barrier for various gas separation utilization without the needs of additional modification, in contrary to previously proposed porous graphene sheets^10, 26^.

## Results

### Energy profiles with ZPE correction

We firstly optimized the unit cell of C_2_N-*h*2D using the density function theory (DFT) implemented in Vienna *Ab initio* Simulation Package (VASP)^27, 28^. The lattice is calculated to be 8.33 Å, in good agreement with the experimental data^29^. Figure 1(a) presents a top view of a fully relaxed (2 × 2) C_2_N-*h*2D supercell. The pore size characterized by the diameter of the inscribed circle is 5.51 Å. In order to regulate the energy barrier of a hydrogen molecule passing through the membrane, a series of biaxial tensile strain, defined by the ratio of the deformation Δ*a* to the initial lattice constant *a*_0_, *ε* = Δ*a*/*a*_*0*_, were then applied to enlarge the pore of the C_2_N-*h*2D membrane. The minimum energy pathway of hydrogen molecule passing through the C_2_N-*h*2D membrane under strains (0%, 2%, 4% and 6%) were searched by the climbing image nudged elastic band method (CNEB)^30, 31^, where the isotropic effect was not involved. Along the diffusion pathway, the displacements of the molecule parallel to the membrane are negligible compared to that perpendicular to the membrane (Fig. S1). We therefore took a pseudo-1D approximation (along the perpendicular direction) to treat the transmission process for simplification. Besides, the quantum effects of the translation, rotation and vibration of the molecule were taken into account through the ZPE correction as discussed below.

The ZPE correction for each image in the CNEB method was calculated on the basis of the density functional perturbation theory implemented in VASP, and then added to the CNEB energy profiles to obtain the effective penetration barriers. Three translational modes, two rotational modes and one vibrational mode were involved in the ZPE correction, among which the vibrational mode dominates. The effective energy barriers for H_2_ and D_2_ are well separated due to different ZPE values, as shown in Fig. 1(b). Each energy profile for both isotopologues consists of two wells separated by a wall. The wells correspond to the adsorption of hydrogen isotopologues on the C_2_N-*h*2D membrane, while the barriers arise from the pore confinement. The effective barriers for H_2_ (solid circles, solid lines) and D_2_ (open circles, dashed lines) through the pore of the membrane decrease with the increase of the tensile strain. Similar to the case in the well confinement^7^, D_2_ always experiences a lower barrier compared with H_2_, due to the smaller ZPE correction values. Additionally, this difference decreases with increasing tensile strain (Fig. S2), indicating that ZPE effect becomes enhanced at high energy barrier conditions.

### Kinetic quantum sieving

**Figure 2:**
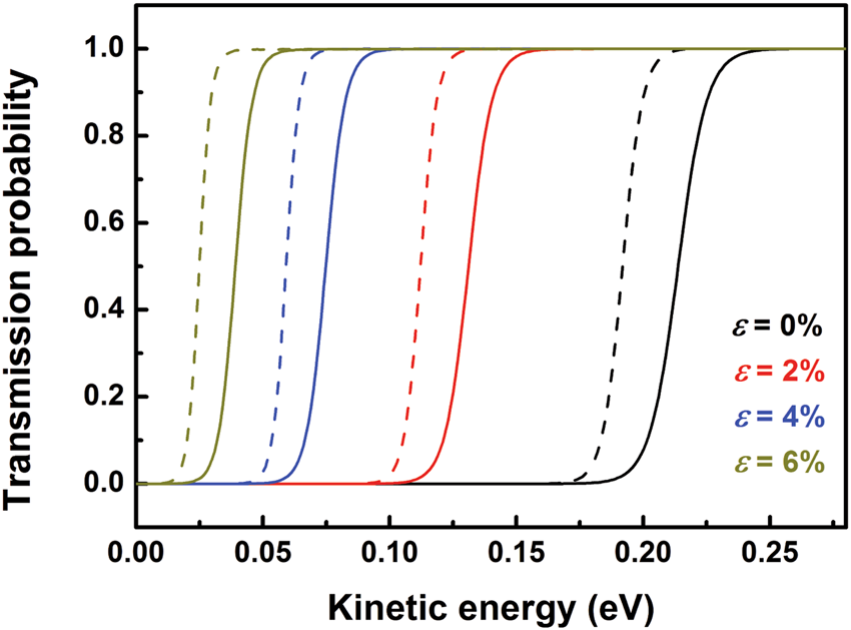
Quantum tunneling probability *t*(*E*) of hydrogen passing through the pore of C_2_N-*h*2D membrane as a function of kinetic energy under different strains. The solid curves represent the quantum-mechanical transmission of H_2_ and the dashed curves represents that of D_2_, respectively.

We utilized the one-dimensional (1D) finite difference method^32^ to evaluate the quantum tunneling probability *t*(*E*) (Fig. 2), the D_2_ permeance *Q*(*D*_*2*_) and the kinetic selectivity *S*(*D*_*2*_/*H*_*2*_)^19, 21, 33, 34^. Unlike the case of helium isotopes^21, 33^, the ZPE correction leads to a lower barrier for D_2_ than that for H_2_, and thus the larger quantum tunneling probability of D_2_.

**Figure 3:**
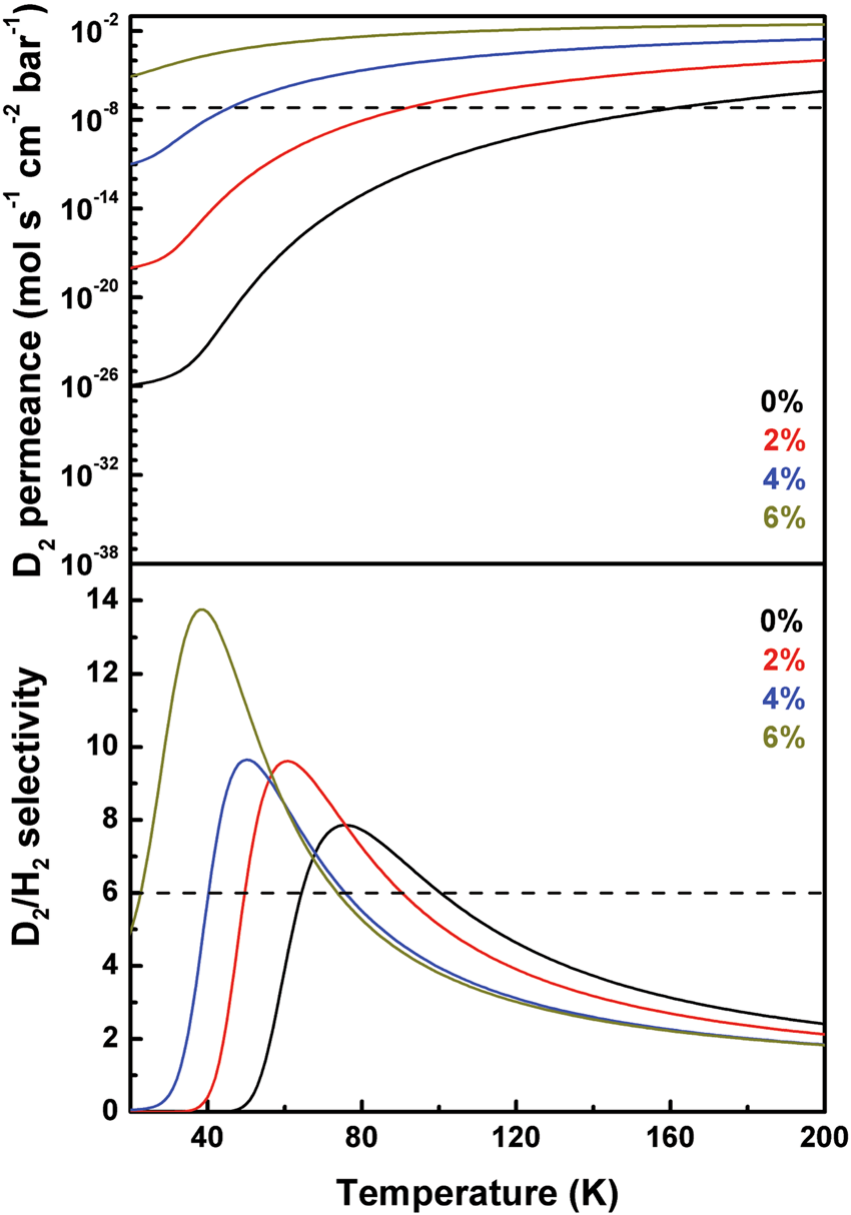
The D_2_ permeance and the kinetic selectivity under different strains for temperature ranging from 20 to 200 K. Different colors indicate different strains ranging from 0% to 6%. The dashed lines indicate the industrial-acceptable values for permeance (6.7 × 10^−8^ mol/s/cm^2^/bar) and selectivity (6), respectively.

As only the pore areas contribute to hydrogen transmission, we defined a fractional parameter in the permeance calculations $\alpha ={\rm{2}}\pi /\sqrt{{\rm{3}}}({R}_{{H}_{2}}^{2}/{a}^{2})$, where ${R}_{{H}_{2}}=2.89$ Å is the kinetic diameter of H_2_ or D_2_ molecule^35^ and *a* is the lattice constant of the strained C_2_N-*h*2D. Therefore, the D_2_ permeance is

1 $$Q({D}_{2})=\frac{\alpha }{\sqrt{2\pi {m}_{{D}_{2}}{k}_{B}T}}p(T)$$

2 $$p(T)=\int p(E,T)t(E)dE$$

3 $$p(E,T)=\frac{1}{\sqrt{4\pi {k}_{B}TE}}{e}^{-E/{k}_{B}T}$$

where *p*(*T*) is the thermally-weighted transmission that has to be numerically evaluated via Eq. (2) and both the incoming pressure and the pressure drop are assumed to be 1 bar. Figure 3 depicts the D_2_ permeance and the kinetic selectivity variation with temperature under different tensile strains. The D_2_ permeance increases with the increase of the tensile strain, as lower barriers permit more flux. The selectivity varies non-monotonically with the temperature: with the increase of temperature, the D_2_/H_2_ kinetic selectivity increases and reaches a maximal value at a critical temperature, above which it drastically decreases with temperature, similar to the results of atomistic molecular dynamics simulations^17^. The critical temperature shifts towards to a lower value with the increase of tensile strain. These variations indicate the competition between the quantum tunneling and the ZPE: at low temperatures, the quantum tunneling effect is dominating which favors the transmission of the lighter isotope, while as temperature increases the heavier isotopes are more inclined to pass through the membrane since ZPE becomes dominating. Under 4% and 6% strains, the membrane exhibit optimized efficiency on both permeance and selectivity, which are 9.6 and 2.6 × 10^−7^ mol/s/cm^2^/bar at 51 K (4% strain), and 13.7 and 2.04 × 10^−4^ mol/s/cm^2^/bar at 39 K (6% strain), respectively. It has been demonstrated that the C_2_N-*h*2D is stable under strain less than 10%^21^, the 4~6% strained membrane is thus practical for industrial application.

It is noteworthy that there are other hydrogen isotopologues, such as HD, HT, DT, T_2_. Using the same strategy, we have calculated the ZPE corrected energy barriers for these hydrogen isotopologues (Fig. S3) and evaluated the separation efficiency between all six isotopes (Fig. S4). The separation abilities of C_2_N-*h*2D membrane for these hydrogen isotopologues are summarized in Table 1. It can be seen that C_2_N-*h*2D membrane is available for separation of H_2_/D_2_, H_2_/DT, H_2_/T_2_, HD/D_2_, HD/DT, HD/T_2_, HT/D_2_, HT/DT, HT/T_2_ with good permeance and selectivity.

**Table 1:**
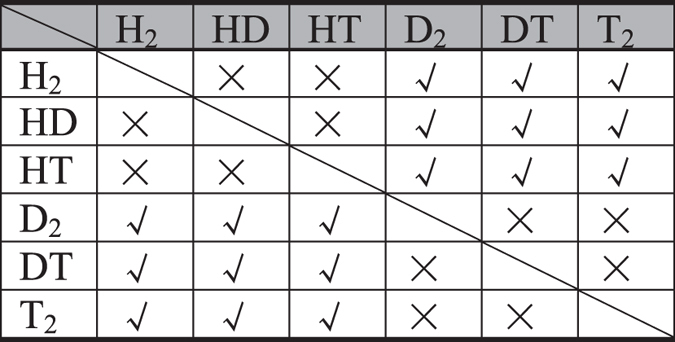
The separation abilities of C_2_N-*h*2D membrane for hydrogen isotopologues (H_2_, HD, HT, D_2_, DT, T_2_). The tick symbol indicates that the C_2_N-*h*2D membrane is able to efficiently separate two isotopologues with industrial acceptable permeance and selectivity, while the cross symbol represents the opposite situation.

### Equilibrium quantum sieving

The thermally-driven equilibrium sieving calculations were performed following the Schrier’s approach^22, 26^, which combines transition state theory (TST) with quantum tunneling effect. This strategy holds for the case where two ideal gases at different temperature (cold reservoir and hot reservoir) are separated by a nanoporous barrier, e.g. C_2_N-*h*2D membrane. Here we took the H_2_/D_2_ separation as an example to investigate the equilibrium quantum sieving efficiency of the C_2_N-*h*2D membrane. As the tunneling barriers for two isotopologues are different in our case, the H_2_/D_2_ separation factor is changed accordingly:

4 $${r}_{TST+Q}=(\frac{{p}^{{D}_{2}}({T}_{H})}{{p}^{{H}_{2}}({T}_{H})})(\frac{{p}^{{H}_{2}}({T}_{C})}{{p}^{{D}_{2}}({T}_{C})})(\frac{{p}_{classical}^{{H}_{2}}({T}_{H})}{{p}_{classical}^{{D}_{2}}({T}_{H})})(\frac{{p}_{classical}^{{D}_{2}}({T}_{C})}{{p}_{classical}^{{H}_{2}}({T}_{C})}){e}^{-({V}_{{H}_{2}}-{V}_{{D}_{2}})/(1/({k}_{B}{T}_{C})-1/({k}_{B}{T}_{H}))}$$

where ${p}_{classical}(T)={\rm{1}}/{\rm{2}}Erfc[\sqrt{V/{k}_{B}T}]$ is the thermally-weighted classical barrier transmission probability and *V* corresponds to the barrier height in Fig. 1(b).

**Figure 4:**
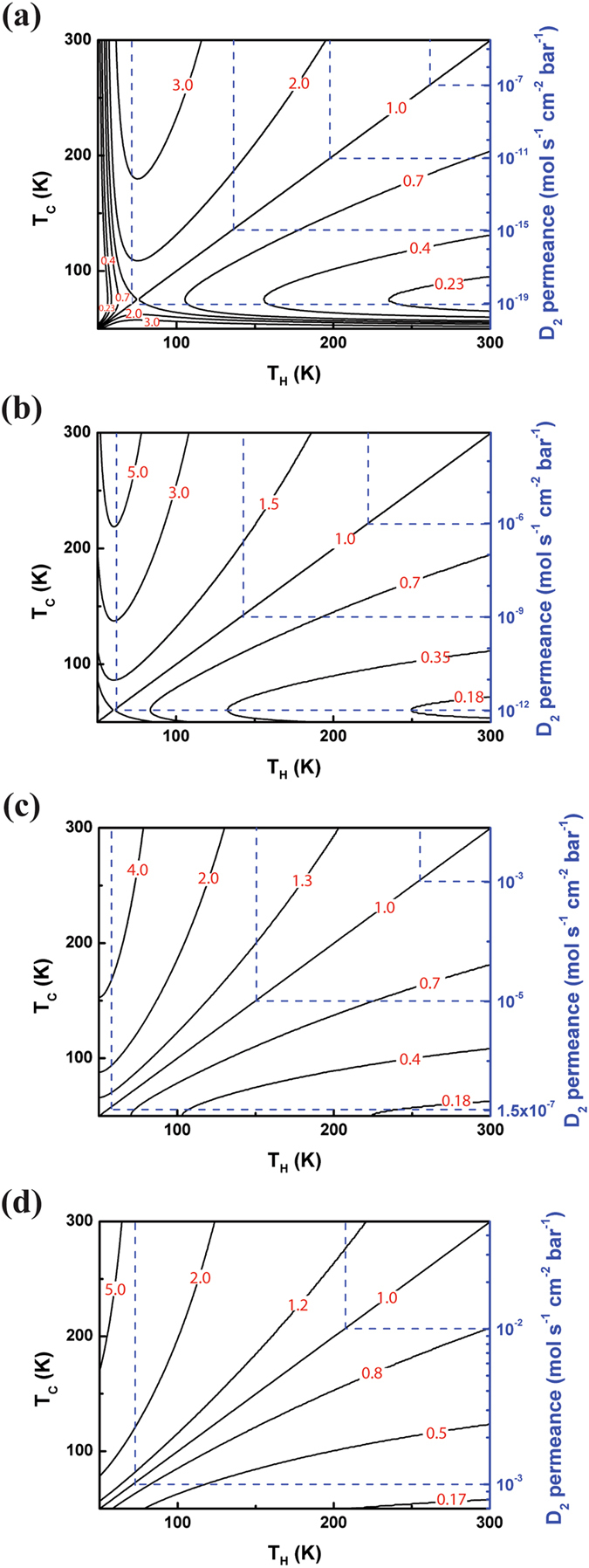
Thermally-driven separation factor *r*_*TST*+*Q*_ indicated by black solid contour at low temperature regime and the corresponding D_2_ permeance by the blue dotted contours labeled on the right axis. (**a**) for strain of 0%, (**b**) for strain of 2%, (**c**) for strain of 4% and (**d**) for strain of 6%.

Figure 4 depicts the H_2_/D_2_ separation factors obtained for the strained C_2_N-*h*2D membrane at thermal reservoir temperatures ranging from 50 K to 300 K. Without strain, the competition between the ZPE and quantum tunneling results in both *r*_*TST+Q*_ > 1 and *r*_*TST+Q*_ < 1 at different reservoir temperature conditions, which means that by properly choosing the reservoir temperature, either H_2_ or D_2_ enrichment can be achieved. However, when strain larger than 2% is imposed to this membrane, only D_2_ enrichment is obtained regardless of mechanisms. Especially, under strain of 6% (Fig. 4(d)), D_2_ enrichment with *r*_*TST+Q*_ = 0.17 (corresponding to 6:1 isotope enrichment) can be obtained with permeance of 8 × 10^−4^ mol/s/cm^2^/bar which meets the requirement of industrial applications.

## Discussion

Different from previous strategies employing potential well confinement for hydrogen isotopologues separation, we here make use of a potential barrier by a 2D membrane. Both kinetic and equilibrium quantum sieving with competitive separation efficiency can be easily achieved in an already synthesized C_2_N-*h*2D membrane under suitable strain, which provides an alternate approach for hydrogen isotopologues separation. More importantly, the C_2_N-*h*2D membrane exhibits intrinsic confinement to the hydrogen isotopologues, without the need of post-modifications or precise control of the material structure during manufacture.

The proper potential barrier provided by the strained C_2_N-*h*2D membrane ensures both high selectivity and high permeance, which also sets up a suitable window for highly-efficient hydrogen isotopologues separation. The barrier-based quantum sieving effect may also hold in other 2D porous membranes for this utility. Furthermore, the strain-tunable quantum sieving of 2D membranes offers a convenient manner for improving separation efficiency under various conditions.

In conclusion, by first-principles calculations, we show that both kinetic and equilibrium quantum sieving for D_2_ enrichment can be obtained via tunneling through a potential barrier in a strained C_2_N-*h*2D membrane. The competition between the ZPE effect and quantum tunneling effect leads to high separation efficiency with industrial acceptable selectivity and permeance. Our results provide an experimentally available 2D porous membrane as a superior quantum sieving that holds promise for both scientific and industrial applications.

## Methods

Our first-principles calculations were performed in the framework of density functional theory (DFT) using the plane-wave pseudopotential approach as implemented in the Vienna *Ab initio* Simulation Package (VASP)^27, 28, 36^. The electron-electron interactions were treated using a generalized gradient approximation (GGA) in the form of Perdew-Burke-Ernzerhof (PBE) for the exchange-correlation functional^36^. The van der Waals (vdW) interactions were included explicitly by using the empirical correction scheme of Grimme (DFT-D2)^37^. The energy cutoff of the plane waves was set to 500 eV with an energy precision of 10^−4^ eV. The atomic coordinates were fully relaxed using a conjugate gradient scheme without any symmetry restrictions until the maximum force on each ion was smaller than 0.02 eV/Å. Vacuum space larger than 15 Å was used to avoid the interaction between adjacent images. The Monkhorst-Pack meshes of 7 × 7 × 1 were used in sampling the Brillouin zone for the 2 × 2 supercells of C_2_N-*h*2D lattice.

The ZPE correction was calculated by the density functional perturbation theory (DFPT) implemented in VASP. In this part of calculation, the energy cutoff of the plane waves was set to 500 eV with an energy precision of 10^−8^ eV and the maximum force on each ion was set to be 0.001 eV/Å.

The quantum tunneling probability calculations were performed using a 1D finite difference method. The grid density was set to be 0.01 Å and a region of 1 Å located 5 Å away from the peak of the barrier was chosen for the incident planewaves, where the barriers is lower than 0.001 eV.

## Electronic supplementary material

Supplementary Material
