# Supplementary material for: Efficient hydrogen isotopologues separation through a tunable potential barrier: The case of a C2N membrane
Source: Sci Rep. 2017 May 3;7:1483. doi: 10.1038/s41598-017-01488-8 (PMC5431156; doi:10.1038/s41598-017-01488-8)
Supplement: Supplementary file 1 — Supplementary Material [file 41598_2017_1488_MOESM1_ESM.pdf]

Supplementary material for

Efficient hydrogen isotopologues separation through a tunable potential barrier: The case of a C_2N membrane

Yuanyuan Qu[a], *Feng Li*[a,b], *Mingwen Zhao*[a*]

[a] *School of Physics, Shandong University, Jinan 250100, Shandong, China;*

[b] *School of Physics and Technology, University of Jinan, Jinan 250022, Shandong, China*

Supplementary Figures

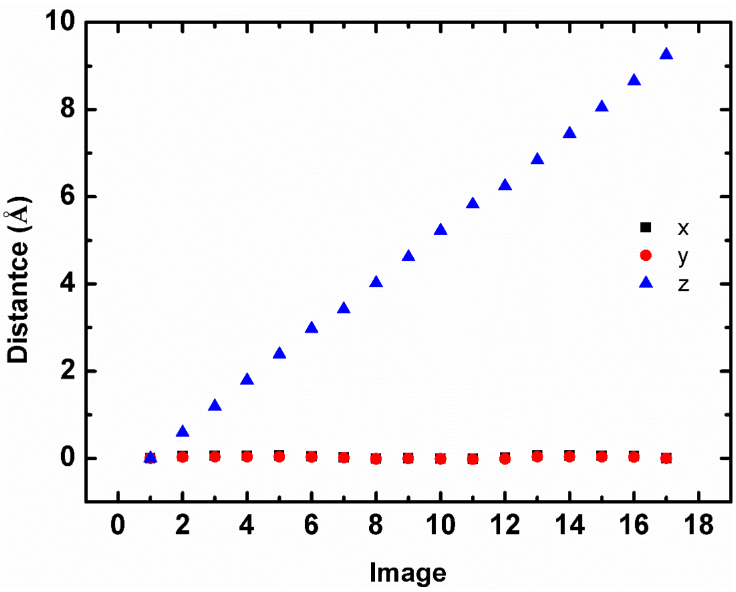

Figure S1 The center of mass displacement of hydrogen molecule passing though the pore of C_2N-*h*2D membrane under no strain. The parallel displacements are x, y directions and the perpendicular displacement is z direction.

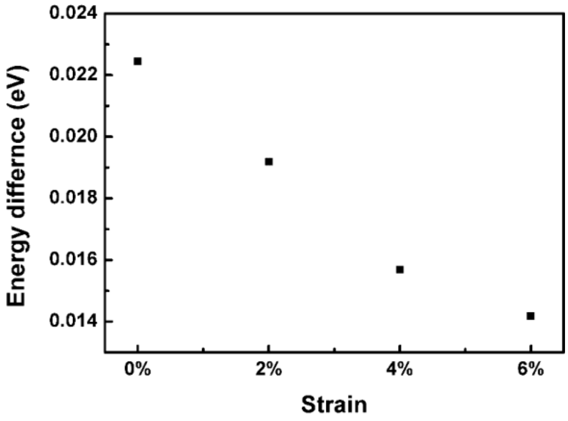

Figure S2 The energy difference in the penetration barriers between H_2 and D_2 at the pore center after taking ZPE correction.

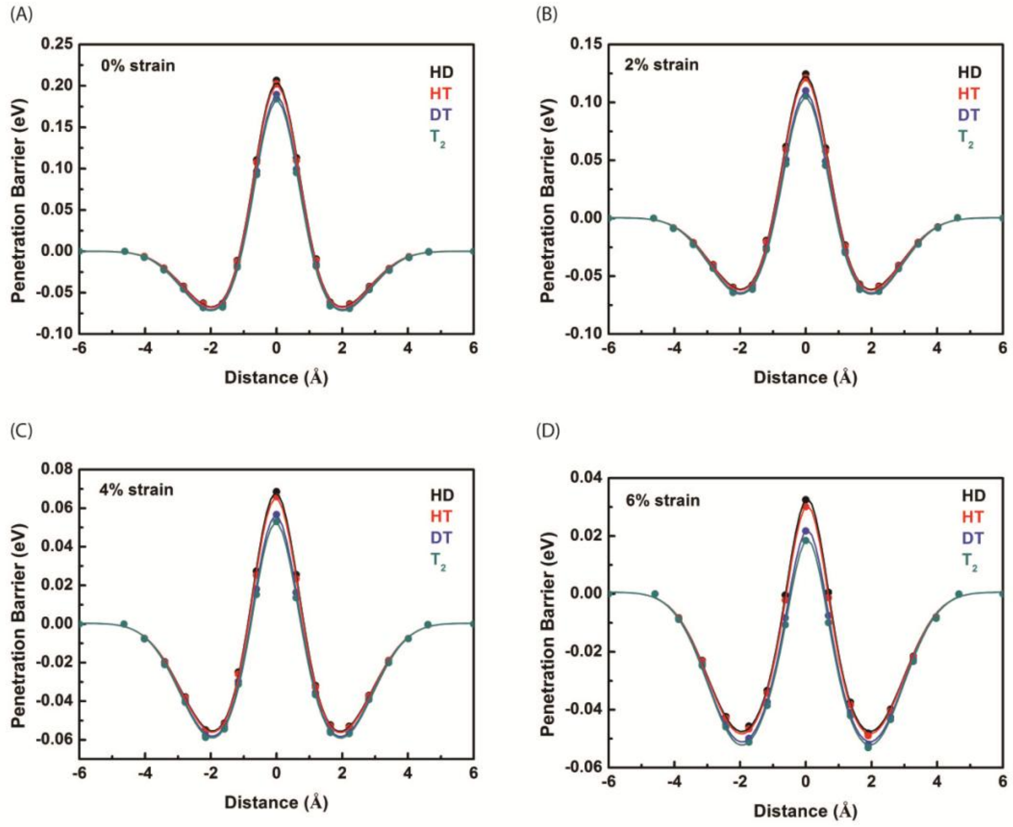

Figure S3. The effective energy barriers of HD, HT, DT, T_2 passing through C_2N-*h*2D membrane along the perpendicular direction under different tensile strains. Colored points indicate the results obtained by the reduced mass approximation ($ZPE_{iso} = ZPE_{H_2}\sqrt{\frac{\mu_{H_2}}{\mu_{iso}}}$); while the curves show the numerically interpolated potentials.

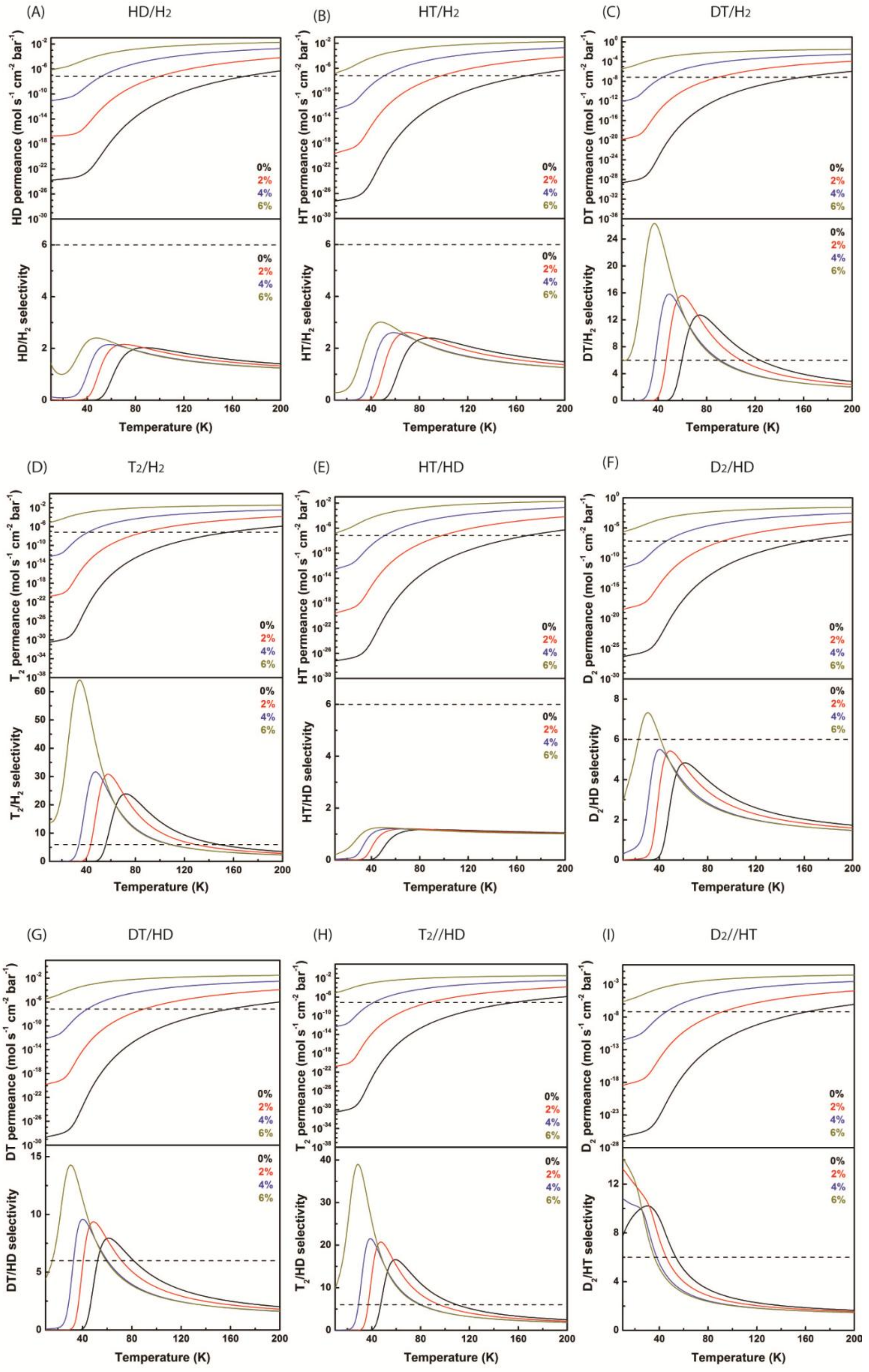
(A) HD/H2
(B) HT/H2
(C) DT/H2
(D) T2/H2
(E) HT/HD
(F) D2/HD
(G) DT/HD
(H) T2//HD
(I) D2//HT
HD permeance (mol s-1 cm-2 bar-1)
HT permeance (mol s-1 cm-2 bar-1)
DT permeance (mol s-1 cm-2 bar-1)
T2 permeance (mol s-1 cm-2 bar-1)
D2 permeance (mol s-1 cm-2 bar-1)
HD/H2 selectivity
HT/H2 selectivity
DT/H2 selectivity
T2/H2 selectivity
HT/HD selectivity
D2/HD selectivity
DT/HD selectivity
T2/HD selectivity
D2/HT selectivity
0%
2%
4%
6%
Temperature (K)

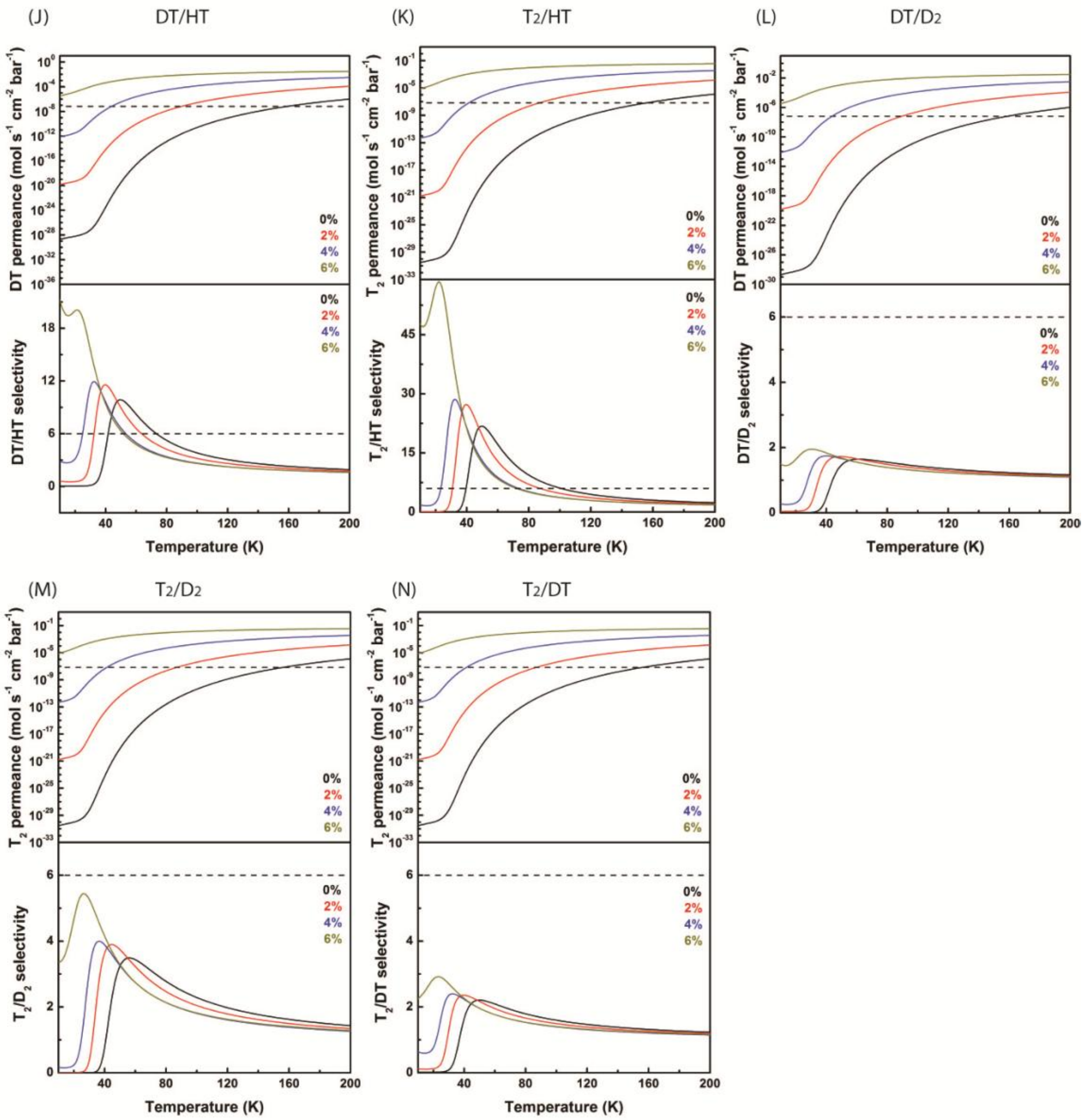

Figure S4. Permeance and the kinetic selectivity under different strains for temperature ranging from 10 to 200 K for difference isotopologues. Different colors indicate different strains ranging from 0% to 6%. The dashed lines indicate the industrial-acceptable values for permeance (6.7 ×10-8 mol/s/cm2/bar) and selectivity (6), respectively.
